# Relations of postural change in blood pressure with hypertension-mediated organ damage in middle-aged adults of the Framingham heart study: A cross-sectional study

**DOI:** 10.3389/fcvm.2022.1013876

**Published:** 2022-11-01

**Authors:** Leroy L. Cooper, Jian Rong, Pauline Maillard, Alexa Beiser, Naomi M. Hamburg, Martin G. Larson, Charles DeCarli, Ramachandran S. Vasan, Sudha Seshadri, Gary F. Mitchell

**Affiliations:** ^1^Department of Biology, Vassar College, Poughkeepsie, NY, United States; ^2^Boston University and NHLBI’s Framingham Study, Framingham, MA, United States; ^3^Department of Neurology and Center for Neurosciences, University of California, Davis, Davis, CA, United States; ^4^Department of Biostatistics, Boston University School of Public Health, Boston, MA, United States; ^5^Department of Neurology, Boston University School of Medicine, Boston, MA, United States; ^6^Evans Department of Medicine, Boston University School of Medicine, Boston, MA, United States; ^7^Whitaker Cardiovascular Institute, Boston University School of Medicine, Boston, MA, United States; ^8^Department of Mathematics and Statistics, Boston University, Boston, MA, United States; ^9^Section of Cardiology, Department of Medicine, Boston University Schools of Medicine, Boston, MA, United States; ^10^Section of Preventive Medicine and Epidemiology, Department of Medicine, Boston, University Schools of Medicine, Boston, MA, United States; ^11^Department of Epidemiology, Boston University School of Public Health, Boston, MA, United States; ^12^Glenn Biggs Institute for Alzheimer’s and Neurodegenerative Diseases, San Antonio, TX, United States; ^13^Cardiovascular Engineering, Inc., Norwood, MA, United States

**Keywords:** epidemiology, postural blood pressure, kidney damage, vascular function, hypertension-mediated organ damage

## Abstract

**Background:**

Dysregulation of compensatory mechanisms to regulate blood pressure (BP) upon postural change is a phenotype of BP variability and an emerging risk factor for cardiovascular outcomes.

**Materials and methods:**

We assessed postural change in BP (starting 2 min after standing from a supine position), carotid-femoral pulse wave velocity (cfPWV), and markers of hypertension-mediated organ damage (HMOD) in the heart, kidney, and brain in Framingham Third Generation, Omni-2, and New Offspring Spouse Cohort participants. We related vascular measures (postural change in BP measures and cfPWV) with HMOD in 3,495 participants (mean age 47 years, 53% women) using multivariable logistic and linear regression models.

**Results:**

In multivariable-adjusted models, we did not observe significant associations of vascular measures with presence of left ventricular hypertrophy, albuminuria, covert brain infarcts, or white matter hyperintensities (Bonferroni-adjusted *P*-values > 0.05/20 > 0.0025). In multivariable models, greater cfPWV (est. β = 0.11 ± 0.03; *P* < 0.001), but not postural change in BP measures (Bonferroni-adjusted *P*-values > 0.05/20 > 0.0025), was associated with higher white matter free water using brain magnetic resonance imaging. In multivariable models, greater postural change in pulse pressure was associated with higher urinary albumin-creatinine ratio (est. β = 0.07 ± 0.02; *P* < 0.001). No other postural change in BP measure was associated with urinary albumin-creatinine ratio (Bonferroni-adjusted *P*-values > 0.05/20 > 0.0025). In sex-specific analyses, higher cfPWV was associated with higher urinary albumin-creatinine ratio in men (est. β: 0.11 ± 0.04; *P* = 0.002) but not in women (est. β: 0.03 ± 0.03; *P* = 0.44). We also observed marginal to strong effect modification by above vs. at/below median postural change in BP for the association of cfPWV with urinary albumin−creatinine ratio (Bonferroni-adjusted interaction *P* < 0.001–0.01). Vascular measures were not related to left ventricular mass index or fractional anisotropy (Bonferroni-adjusted *P*-values > 0.05/20 > 0.0025).

**Conclusion:**

Baroreflex dysfunction is associated with greater subclinical kidney damage. Additionally, relations of higher aortic stiffness with greater kidney damage may be modified by associated baroreflex dysregulation.

## Introduction

Blood pressure responses to postural maneuvers is a phenotype of blood pressure variability and an emerging risk indicator for hypertension-mediated organ damage (HMOD) and cardiovascular disease (CVD) (1). Upon standing from a supine position, baroreceptors in the aortic arch and carotid sinus sense the initial drop in systemic pressure and induce a compensatory increase in heart rate and peripheral vascular resistance, which quickly reestablishes mean arterial pressure (MAP) (2). Traditional approaches that categorize pathologic phenotypes of blood pressure dysregulation upon standing as either orthostatic hypotension or hypertension conflate the effects of various blood pressure measures and confound interpretation of observed relations. However, assessment of more subtle, non-pathologic changes in individual blood pressure measures across the full range of normal responses upon standing may be more informative in elucidating the nuanced pathophysiology underlying the observed associations of alterations in the baroreflex or other regulatory pathways with adverse outcomes.

Prior studies revealed that elevated aortic stiffness may contribute to dysregulation of postural changes in blood pressure (3–7). Elevated aortic stiffness is characterized by blunted microvascular reactivity (8) and impedance matching of the elastic and muscular arteries, which may transmit potentially harmful pulsatile energy into the microcirculation, leading to HMOD (9, 10). For example, we showed that elevated aortic stiffness is associated with a blunted increase in MAP upon standing in younger Framingham Heart Study (FHS) participants (4). Additionally, in a similar sample, we showed that brain volume is particularly susceptible to postural change in MAP but primarily among participants with higher levels of aortic stiffness (11). Yet, the inter-relations of postural change in various blood pressure measures, aortic stiffness, and additional indicators of HMOD have not been examined. Based on the foregoing observations, associations of aortic stiffness with microvascular HMOD may differ by extent of baroreflex dysregulation.

We aimed to assess cross-sectional relations of CVD risk factors with postural change (supine to standing) in blood pressure and the relations of postural change in blood pressure and aortic stiffness with preclinical markers of HMOD of the heart, kidney, and brain. We hypothesize that postural change in blood pressure is associated with preclinical markers of HMOD. Additionally, we hypothesized that the degree of postural change in blood pressure modifies the associations of aortic stiffness with HMOD.

## Materials and methods

### Study sample

The study sample was drawn from the Framingham Third Generation, Omni-2, and New Offspring Spouse Cohorts, which have been described previously (12, 13). Omni participants were diverse, non-White individuals living in the Boston metro-west area, and New Offspring Spouse participants (not previously enrolled in the Offspring Cohort) were parents of Third Generation participants. Central hemodynamics, brain magnetic resonance imaging (MRI), and urinalysis for kidney albumin excretion were performed at the second examination cycle (2008–2011). We used participants’ transthoracic echocardiography data, which was performed at the first examination cycle (2002–2005). In total, 3,800 participants were eligible for this investigation. Participants were excluded for the following reasons: prevalent peripheral artery disease (*n* = 42); missing covariate information (*n* = 92); incomplete tonometry or hemodynamic assessment (*n* = 171). Written informed consent was obtained from all study participants, and the research protocols were approved by the Institutional Review Board at Boston University School of Medicine.

### Hemodynamic assessment with arterial tonometry

Hemodynamic assessment was performed as previously described (14, 15). Following 5 min of rest, we measured auscultatory blood pressure in the right arm using a standard upper arm cuff in the supine position. We obtained non-invasive arterial tonometry with simultaneous electrocardiography from brachial, radial, femoral, and carotid arteries using a custom tonometer. For blinded analyses, we digitized and transferred tonometric data to the core laboratory (Cardiovascular Engineering, Inc., Norwood, MA). Using the electrocardiographic R-wave, we signal-averaged and synchronized tonometry waveforms (15). We calculated supine MAP as the integral of the signal-averaged brachial pressure tonometry waveform, which was calibrated by using the auscultatory systolic and diastolic cuff pressures. We calculated carotid-femoral pulse wave velocity (cfPWV), the reference standard for aortic stiffness, from tonometry waveforms and body surface measurements that adjusted for parallel transmission in the aortic arch and brachiocephalic artery using the suprasternal notch as a fiducial point (9).

We assessed postural change in blood pressures—MAP, systolic blood pressure (SBP), diastolic blood pressure (DBP), and pulse pressure (PP)—as previously described (4, 11). We acquired a second auscultatory blood pressure measurement in the standing position after the hemodynamic acquisition and starting 2 min after standing from a supine position. We used the brachial cuff oscillometric signal recorded for 20 s. We defined postural change in blood pressure as the difference in blood pressure value while standing vs. blood pressure value while supine. A negative value for postural change in blood pressure indicates a drop in blood pressure upon standing.

### Hypertension-mediated organ damage

We performed brain MRI on participants to ascertain the presence of covert brain infarcts and white matter hyperintensities (16, 17). We defined the presence of large (extensive) white matter hyperintensities in cases where the natural log of the ratio of white matter hyperintensity volume to total cranial volume was >1 standard deviation above the age-adjusted mean value (18). We manually identified the presence of covert brain infarcts based on their size, location, and imaging characteristics as previously described (18). We used diffusion tensor imaging to assess white matter free water content and fractional anisotropy, which are sensitive indicators of brain parenchymal white matter integrity, using previously described algorithms (19, 20). We assessed the segmentation of white matter hyperintensities and total cranial volume from fluid-attenuated inversion recovery and T1-weighted images using automated procedures as previously described (21, 22). We coregistered free water, fractional anisotropy, and white matter hyperintensities maps to a minimum deformation template for analyses; each voxel in the coregistered multimodal volumetric images corresponded to the same location in the brain across individuals. Operators blinded to participants’ demographic and clinical data assessed the MRI and diffusion tensor imaging images.

We measured urinary albumin-creatinine ratio (UACR) using spot morning urine samples obtained from participants at the exam visit. We assessed urinary albumin concentration using an immunoturbidimetry assay and measured urinary creatinine using a modified Jaffé method. We defined the presence of albuminuria using sex-specific cutpoints for UACR of ≥17 mg/g for men and ≥25 mg/g for women (23). UACR is strongly correlated with albumin excretion rates obtained from 24-h urine collection and reliably assesses urinary albumin excretion (24).

We performed transthoracic echocardiography, and an experienced sonographer or cardiologist evaluated all echocardiograms using a standardized reading protocol as previously described (25). We calculated left ventricular (LV) mass using cardiac dimensions quantified using digital images and the leading-edge technique, and we indexed LV mass to body surface area according to American Society of Echocardiography guidelines (26). We defined the presence of LV hypertrophy using sex-specific mass indices of >95 g/m^2^ for women and >115 g/m^2^ for men (26, 27).

### Clinical evaluation and covariates

We performed a routine assessment of medical history, physical examination, and electrocardiography at each examination (28). We assessed height and weight during the examination and calculated body mass index by dividing weight in kilograms by the square of the height in meters. We assessed age, sex, use of antihypertensive and hyperlipidemia medications, smoking (current vs. non-smoker), and alcohol consumption via questionnaires. We measured serum lipid levels from a fasting blood test. Criteria for diabetes mellitus were a fasting glucose ≥126 mg/dL (7.0 mmol/L) or treatment with insulin or an oral hypoglycemic agent. To assess the presence of atrial fibrillation, we reviewed all available electrocardiograms from examination cycles, outpatient and inpatient hospital records, or ambulatory electrocardiogram monitoring. History of CVD was defined as a history of coronary heart disease, congestive heart failure, stroke, or transient ischemic attack.

### Statistical analyses

Demographic, hemodynamic, and HMOD summary data and characteristics of the sample were tabulated. cfPWV was inverted to limit heteroscedasticity; the inverted value was multiplied by −1,000 to convert units to ms/m and rectify the directionality of the association with aortic stiffness. White matter hyperintensity volumes were natural log-transformed to reduce skewness. All continuous outcome measures were standardized (mean 0, SD 1) for the modeling.

We assessed relations of postural change in blood pressure measures with various CVD risk factors using stepwise multivariable regression models that adjusted for age, sex, and corresponding supine blood pressure; the threshold value for inclusion and removal from the model was *P* < 0.05. CVD risk factors were selected *a priori* based on literature review and included body mass index, heart rate, fasting glucose, triglycerides, HbA1c, total/high-density lipoprotein cholesterol ratio, hypertension treatment, hyperlipidemia treatment, diabetes mellitus, alcohol consumption (past year), smoking status, prevalent CVD, and prevalent atrial fibrillation.

We used multivariable logistic regression to analyze the presence of each dichotomous HMOD variable (LV hypertrophy, albuminuria, covert brain infarcts, and white matter hyperintensities) related to the postural change in blood pressure. Models were adjusted for age, sex, corresponding supine blood pressure, and all CVD risk factors significantly related to any postural change in the blood pressure measures in the stepwise models. Expanded models additionally adjusted for cfPWV. We assessed potential effect measure modification by sex, age (quartiles 1–3 vs. quartile 4), and hypertension treatment by incorporating corresponding interaction terms in the logistic regression models. We tested for non-linear associations using restricted cubic spline models to evaluate associations of postural change in MAP and PP with log odds of covert brain infarcts and white matter hyperintensities in models adjusting for the clinical covariates described above. We placed knots at the 5th, 33rd, 67th, and 95th percentiles of postural change in blood pressures.

Furthermore, we assessed the relations of cfPWV and postural change in blood pressure measures with continuous measures of HMOD (LV mass index, UACR, free water, and fractional anisotropy) using multivariable linear regression adjusted for age, sex, corresponding supine blood pressures, and all CVD risk factors that were significantly related to any measure of postural change in blood pressure in the stepwise models. Supine MAP was a covariate in models where cfPWV was the independent variable of interest. We assessed potential effect measure modification by sex, age (quartiles 1–3 vs. quartile 4), and hypertension treatment by incorporating corresponding interaction terms in the linear regression models. We further assessed potential effect measure modification by postural change in blood pressure (above median change vs. at/below median) on the observed relations of cfPWV with continuous HMOD indicators by incorporating corresponding interaction terms in the statistical models. We estimated Pearson partial correlations to assess associations between postural changes in heart rate and postural changes in blood pressure measures.

We performed all analyses with SAS version 9.4 for Windows (SAS Institute, Cary, NC). Two-tailed *P* < 0.05 were considered statistically significant for the initial cross-sectional risk factor model for stepwise variable selection. For the primary analyses, Bonferroni-adjusted 2-sided *P*-values (α*_*Bon*_* = 0.05 for primary analyses; α*_*Bon*_* = 0.1 for interaction analyses) were used to assesses statistical significance to adjust for multiple testing.

## Results

**FIGURE 1:**
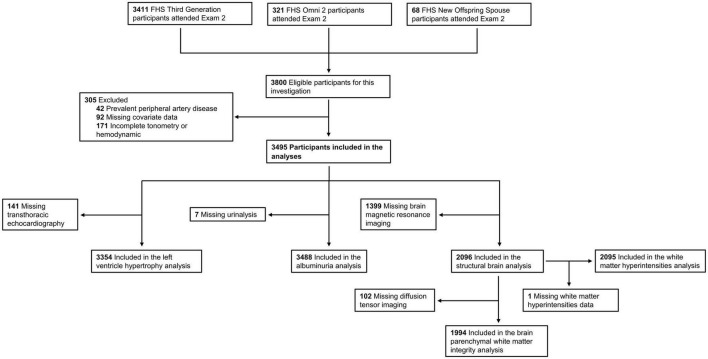
Flow diagram of the analysis sample selection.

We included 3,495 participants [1,844 (53%) women] in the analyses. A flow chart for the study is presented in Figure 1, and characteristics of the study participants are presented in Table 1. Features of vascular measures and HMOD variables are shown in Table 2, and the distributions for postural change in blood pressures measures are presented in the Supplementary Figure 1. Clinical correlates of postural change in blood pressure measures are presented in Supplementary Table 1. Higher age, corresponding supine SBP, and female sex were associated with a lower postural change in SBP. Higher body mass index and heart rate, prevalent alcohol consumption, and smoking were associated with a greater postural change in SBP. Higher age and corresponding supine DBP as well as female sex and prevalent diabetes were associated with a lower postural change in DBP. Higher body mass index, heart rate, triglycerides, and prevalent alcohol consumption were associated with a greater postural change in DBP. Higher age, corresponding supine MAP, and prevalent CVD were associated with a lower postural change in MAP. Higher body mass index, heart rate, total/HDL cholesterol ratio, female sex, prevalent alcohol consumption, and smoking were associated with a greater postural change in MAP. Higher corresponding supine PP and female sex were associated with a lower postural change in PP. Higher age, body mass index, heart rate, and prevalent smoking were associated with a greater postural change in PP.

**TABLE 1:** Clinical characteristics of the sample (*N* = 3,495).

Variable	Value*
Age, years	47 ± 10
Women, n (%)	1,844 (53)
Omni-2 cohort, n (%)	297 (8)
Body mass index, kg/m^2^	27.7 ± 5.4
Total/high-density lipoprotein cholesterol ratio	3.4 ± 1.1
Triglycerides, mg/dL	111 ± 70
Fasting glucose, mg/dL	96 ± 17
Hemoglobin A1c,%	5.5 ± 0.5
Alcohol consumption in the past year, n (%)	2,742 (78)
Hypertension treatment, n (%)	586 (17)
Lipid disorder treatment, n (%)	556 (16)
Prevalent diabetes mellitus, n (%)	164 (5)
Current smoker, n (%)	328 (9)
Prevalent cardiovascular disease, n (%)	45 (1)
Prevalent atrial fibrillation, n (%)	40 (1)

*Values are mean ± standard deviation or number (%).

**TABLE 2:** Vascular measures and hypertension-mediated organ damage variables.

Variable	Value (N = varies)*
**Vascular measures^†^**	
Supine systolic blood pressure, mm Hg	120.8 ± 14.7
Supine diastolic blood pressure, mm Hg	63.0 ± 9.2
Supine mean arterial pressure, mm Hg	87.0 ± 10.8
Supine pulse pressure, mm Hg	57.7 ± 12.1
Supine heart rate, bpm	63 ± 10
Change in systolic blood pressure upon standing, mm Hg	1.5 ± 9.9
Change in diastolic blood pressure upon standing, mm Hg	9.2 ± 7.8
Change in mean arterial pressure upon standing, mm Hg	6.5 ± 7.7
Change in pulse pressure upon standing, mm Hg	−7.7 ± 10.6
Change in heart rate upon standing, bpm	16 ± 9
Carotid-femoral pulse wave velocity, m/s	7.1 ± 1.5
**Hypertension-mediated organ damage dichotomous variables**	
Left ventricular hypertrophy, n/N (%)^‡^	190/3,354 (6)
Albuminuria, n/N (%)	185/3,488 (5)
Covert brain infarcts, n/N (%)	92/2,096 (4)
White matter hyperintensities, n/N (%)	162/2,095 (8)
**Hypertension-mediated organ damage continuous variables**	
Left ventricle mass (indexed to body surface area), g/m^2^ (*N* = 3,354)	83 ± 16
Urinary albumin-creatinine ratio, mg/g (*N* = 3,488)	3.9 (2.5, 7.4)
White matter fractional anisotropy (*N* = 1,994)	0.62 ± 0.01
White matter free water content (*N* = 1,994)	0.20 ± 0.02

*Data are presented as mean ± standard deviation or median (25th, 75th percentile range) unless otherwise noted. Note that N varies (1,994–3,495) for participants based on availability of data.

^†^The supine blood pressure measures are compared to the corresponding change in blood pressure upon standing.

^‡^We defined hypertrophy using sex-specific cutpoints for LV mass index (>95 g/m^2^ for women and >115 g/m^2^ for men).

Relations of vascular measures (cfPWV and postural change in blood pressure measures) with presence of HMOD are presented in Table 3. In multivariable models, we did not observe significant associations of vascular measures with presence of HMOD. We further adjusted models of postural change in blood pressure with presence of HMOD for cfPWV (Supplementary Table 2), and the results were similar. In secondary spline analyses, we did not observe non-linear associations (data not shown). We did not observe interactions by sex, age, or hypertension treatment status for the relations of postural change in blood pressure with the presence of HMOD outcomes. We observed a significant sex interaction for the association of cfPWV with presence of albuminuria (*P* < 0.001) but not for other HMOD outcomes (Supplementary Table 3). However, in stratified analyses, the association of cfPWV with presence of albuminuria was not statistically significant in men (*P* = 0.1) or women (*P* = 0.53). We did not observe interactions by age or hypertension treatment status for the relation of cfPWV with presence of HMOD outcomes.

**TABLE 3:** Multivariable-adjusted relations of postural change in blood pressure and carotid-femoral pulse wave velocity with presence of hypertension-mediated organ damage.

Vascular measures	Left ventricle hypertrophy (*N* = 3,354)		Albuminuria (*N* = 3,488)		Covert brain infarcts (*N* = 2,096)		White matter hyperintensities (*N* = 2,095)	
				
	OR (95% CI)	*P*	OR (95% CI)	*P*	OR (95% CI)	*P*	OR (95% CI)	*P*
ΔSBP	1.18 (1.02, 1.38)	0.03	1.12 (0.96, 1.31)	0.15	1.22 (0.97, 1.52)	0.09	1.05 (0.88, 1.25)	0.6
ΔDBP	1.04 (0.89, 1.22)	0.6	1.03 (0.88, 1.21)	0.7	1.22 (0.97, 1.54)	0.09	1.01 (0.84, 1.20)	0.95
ΔMAP	1.16 (0.98, 1.36)	0.08	1.12 (0.95, 1.32)	0.19	1.28 (1.01, 1.62)	0.045	1.07 (0.89, 1.28)	0.5
ΔPP	1.17 (1.01, 1.36)	0.04	1.10 (0.94, 1.29)	0.2	1.05 (0.84, 1.32)	0.7	1.06 (0.89, 1.27)	0.5
cfPWV	0.99 (0.79, 1.25)	0.9	1.07 (0.85, 1.35)	0.6	1.07 (0.77, 1.48)	0.7	1.45 (1.13, 1.86)	0.004

Odds ratios (OR) expressed per 1 standard deviation higher value of postural change in blood pressure measure. Bonferroni-adjusted *P*-values (*P* = 0.05/20 = 0.0025) were used to assess significance of associations. CI, confidence interval; ΔSBP, postural change in systolic blood pressure; ΔDBP, postural change in diastolic blood pressure; ΔMAP, postural change in mean arterial pressure; ΔPP, postural change in pulse pressure; cfPWV, carotid-femoral pulse wave velocity. All models adjusted for age, sex, corresponding supine blood pressure, body mass index, heart rate, total/high-density lipoprotein cholesterol ratio, alcohol consumption (past year), prevalent cardiovascular disease, current smoker, triglycerides, and prevalent diabetes. Covert brain infarcts and white matter hyperintensities models were further adjusted for total cranial volume and time between tonometry and brain magnetic resonance imaging.

Relations of vascular measures (cfPWV and postural change in blood pressure measures) with individual continuous measures of HMOD are presented in Table 4. In multivariable models, greater postural change in PP (est. β = 0.07 ± 0.02; *P* < 0.001) was associated with higher UACR. Other postural change in blood pressure measures were not associated with UACR. We did not observe sex interactions for the association of postural change in blood pressure with continuous HMOD outcomes (Supplementary Table 4). However, we observed a significant sex interaction for the association of cfPWV with UACR (interaction *P* < 0.001), and in sex-specific analyses, higher cfPWV was associated with higher UACR in men (est. β: 0.11 ± 0.04; *P* = 0.002) but not in women (est. β: 0.03 ± 0.03; *P* = 0.44). In multivariable models, greater cfPWV (est. β = 0.11 ± 0.03; *P* < 0.001) was associated with higher brain free water. No other vascular measure was associated with brain free water. Vascular measures were not related to LV mass index or fractional anisotropy. We did not observe interactions by age or hypertension treatment status for the relations of postural change in blood pressure measures and cfPWV with the continuous HMOD outcomes.

**TABLE 4:** Multivariable-adjusted relations of postural change in blood pressure and carotid-femoral pulse wave velocity with continuous measures of hypertension-mediated organ damage.

Vascular measures	Left ventricular mass index (*N* = 3,354)		UACR (*N* = 3,488)		Free water (*N* = 1,994)		Fractional anisotropy (*N* = 1,994)	
	Est. β ± SE	*P*	Est. β ± SE	*P*	Est. β ± SE	*P*	Est. β ± SE	*P*
ΔSBP	0.04 ± 0.02	0.008	0.05 ± 0.02	0.004	0.03 ± 0.02	0.2	–0.01 ± 0.02	0.8
ΔDBP	0.02 ± 0.02	0.2	–0.01 ± 0.02	0.4	–0.02 ± 0.02	0.3	–0.01 ± 0.02	0.7
ΔMAP	0.04 ± 0.02	0.02	0.03 ± 0.02	0.06	0.02 ± 0.02	0.5	–0.01 ± 0.02	0.5
ΔPP	0.03 ± 0.02	0.03	0.07 ± 0.02	<0.001	0.05 ± 0.02	0.04	0.01 ± 0.02	0.8
cfPWV	–0.04 ± 0.02	0.1	0.06 ± 0.02	0.01	0.11 ± 0.03	<0.001	–0.02 ± 0.03	0.5

UACR, urinary albumin-creatinine ratio; ΔSBP, postural change in systolic blood pressure; ΔDBP, postural change in diastolic blood pressure; ΔMAP, postural change in mean arterial pressure; ΔPP, postural change in pulse pressure; cfPWV, carotid-femoral pulse wave velocity. The regression coefficients (β) are per 1 standard deviation unit increase in vascular measure. Bonferroni-adjusted *P*-values (*P* = 0.05/20 = 0.0025) were used to assess significance of associations. Each vascular measure is considered separately in the models. All models adjusted for age, sex, corresponding supine blood pressure (cfPWV models were adjusted for supine MAP), body mass index, heart rate, total/high-density lipoprotein cholesterol ratio, alcohol consumption (past year), prevalent cardiovascular disease, current smoker, triglycerides, and prevalent diabetes. Free water and fractional anisotropy models were further adjusted for total cranial volume and time between tonometry and brain magnetic resonance imaging.

**FIGURE 2:**
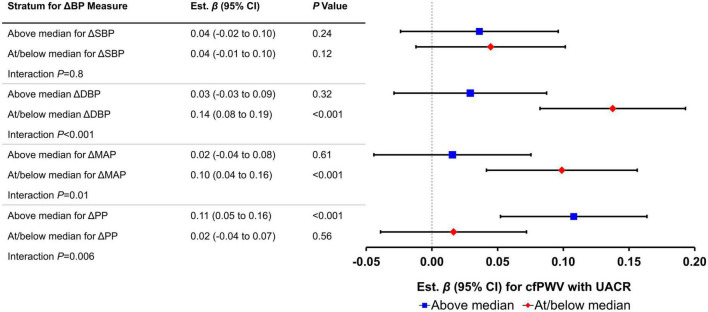
Relations of carotid-femoral pulse wave velocity (cfPWV) with urinary albumin-creatinine ratio (UACR) by median postural change in blood pressure measures (*N* = 3,488). Effect sizes (βs) and 95% confidence intervals are from linear regression models; Bonferroni-adjusted *P*-values (*P* = 0.05/8 = 0.00625) were used to assess significance of associations. Each vascular measure is considered separately in the models. Estimates represent difference in natural log-transformed UACR (in standard deviation units) per 1 standard deviation unit increase in the negative inverse of cfPWV. ΔSBP, postural change in systolic blood pressure; ΔDBP, postural change in diastolic blood pressure; ΔMAP, postural change in mean arterial pressure; ΔPP, postural change in pulse pressure. All models adjusted for age, sex, corresponding supine blood pressure, body mass index, heart rate, total/high-density lipoprotein cholesterol ratio, alcohol consumption (past year), prevalent cardiovascular disease, current smoker, triglycerides, and prevalent diabetes.

In Table 5, we present an assessment of effect modification by postural change in blood pressure for the relation of cfPWV with continuous measures of HMOD. We observed a significant interaction by the above median postural change in PP (*P* < 0.001) and DBP (*P* = 0.006) and marginally significant interaction above median postural change in MAP (*P* = 0.01) for the relation of cfPWV with UACR. Figure 2 depicts the slope of UACR vs. (negative inverse) cfPWV estimated for participants who were above vs. at/below median postural change in blood pressure measures. Among participants who were at or below median postural change in MAP (est. β = 0.10; 95% CI: 0.04–0.16; *P* < 0.001) and DBP (est. β = 0.14; 95% CI: 0.08–0.19; *P* < 0.001) or above median postural change in PP (est. β = 0.11; 95% CI: 0.05–0.16; *P* < 0.001), higher cfPWV was associated with higher UACR. Interactions by the above median postural change in blood pressures for relations of cfPWV with UACR were attenuated upon further stratification by sex (Supplementary Table 5). We did not observe differences in the relation of cfPWV with UACR by the above median postural change in SBP. Interrelations of postural change in heart rate and postural change in blood pressure measures are presented in Supplementary Table 6.

**TABLE 5:** Effect modification by postural change in blood pressure for the relation of aortic stiffness with continuous measures of hypertension-mediated organ damage.

Vascular measures	Left ventricular mass index (*N* = 3,354)		UACR (*N* = 3,488)		Free water (*N* = 1,994)		Fractional anisotropy (*N* = 1,994)	
				
	Est. β ± SE	*P*	Est. β ± SE	*P*	Est. β ± SE	*P*	Est. β ± SE	*P*
cfPWV	**–**0.07 ± 0.03	0.005	0.04 ± 0.03	0.12	0.11 ± 0.04	0.003	–0.04 ± 0.03	0.3
Above median ΔSBP	0.05 ± 0.03	0.1	0.08 ± 0.03	0.02	0.04 ± 0.04	0.4	–0.03 ± 0.04	0.4
*cfPWV × above median ΔSBP	**0.03 ± 0.03**	**0.3**	**–0.01 ± 0.03**	**0.8**	**–0.01 ± 0.04**	**0.8**	**0.03 ± 0.04**	**0.4**
cfPWV	–0.01 ± 0.02	0.7	0.14 ± 0.03	<0.001	0.11 ± 0.04	0.001	–0.05 ± 0.03	0.1
Above median ΔDBP	0.06 ± 0.03	0.03	–0.05 ± 0.03	0.1	–0.08 ± 0.04	0.62	0.01 ± 0.04	0.9
*cfPWV × above median ΔDBP	**–0.005 ± 0.03**	**0.9**	**–0.11 ± 0.03**	**<0.001**	**–0.01 ± 0.04**	**0.9**	**0.08 ± 0.04**	**0.04**
cfPWV	–0.04 ± 0.03	0.08	0.10 ± 0.03	<0.001	0.11 ± 0.04	0.004	–0.05 ± 0.03	0.1
Above median ΔMAP	0.06 ± 0.03	0.04	0.08 ± 0.03	0.02	0.03 ± 0.04	0.5	–0.02 ± 0.04	0.6
*cfPWV × above median ΔMAP	**0.01 ± 0.03**	**0.7**	**–0.08 ± 0.03**	**0.01**	**0.02 ± 0.04**	**0.7**	**0.07 ± 0.04**	**0.08**
cfPWV	–0.01 ± 0.02	0.6	0.02 ± 0.03	0.5	0.09 ± 0.04	0.01	–0.002 ± 0.03	0.9
Above median ΔPP	0.03 ± 0.03	0.3	0.14 ± 0.03	<0.001	0.03 ± 0.04	0.4	0.02 ± 0.04	0.6
*cfPWV × above median ΔPP	**0.01 ± 0.03**	**0.6**	**0.09 ± 0.03**	**0.006**	**0.02 ± 0.04**	**0.6**	**0.01 ± 0.04**	**0.9**

UACR, urinary albumin-creatinine ratio; cfPWV, carotid-femoral pulse wave velocity; ΔSBP, postural change in systolic blood pressure; ΔDBP, postural change in diastolic blood pressure; ΔMAP, postural change in mean arterial pressure; ΔPP, postural change in pulse pressure. cfPWV, above median postural change in blood pressure measure (0 if ≤ median, 1 if > median), and the interaction term (cfPWV × above median change in blood pressure) were entered simultaneously as predictors in the models. The regression coefficients (β) are per 1 standard deviation unit increase in predictor value. *Bonferroni-adjusted *P*-values (*P* = 0.1/16 = 0.0063) were used to assess significance of interaction terms. All models adjusted for age, sex, corresponding supine blood pressure, body mass index, heart rate, total/high-density lipoprotein cholesterol ratio, alcohol consumption (past year), prevalent cardiovascular disease, current smoker, triglycerides, and prevalent diabetes. Free water and fractional anisotropy models were further adjusted for total cranial volume and time between tonometry and brain magnetic resonance imaging. Bold values represent interaction terms.

## Discussion

### Principal findings

We investigated the cross-sectional relations of postural change in blood pressure measures (indicators of baroreflex function) and cfPWV (reference standard for aortic stiffness) with dichotomous and continuous indicators of HMOD in a sample of middle-aged FHS participants. Differences in postural response in pressure pulsatility were associated with greater subclinical kidney damage, and higher levels of aortic stiffness were associated with higher levels of subclinical kidney damage in men. In addition, higher aortic stiffness was associated with greater subclinical cerebral injury. We observed significant effect modification by the above median postural change in blood pressure measures for the relation of aortic stiffness with subclinical kidney damage. Among participants with postural blood pressure responses above the median for MAP and DBP, but at or below the median for PP, the observed relation of higher aortic stiffness with kidney damage was substantially lower. Our results indicate that differences in baroreflex function, as indicated by a blunted increase in MAP and DBP on standing, are associated with greater kidney damage in the presence of a stiffer aorta.

### Postural change in blood pressure measures and hypertension-mediated organ damage

Prior population-based studies suggest that impairment of compensatory mechanisms that regulate blood pressure upon standing is associated with cardiovascular events and adverse outcomes to downstream organs. Although most studies focus on orthostatic hypotension, orthostatic hypertension has been less studied but is emerging as a novel risk factor for CVD events and HMOD (1, 29–33). For example, recent evidence from the SPRINT Study suggests that the prevalence of orthostatic hypertension may be similar to that of orthostatic hypotension (34). Although the criteria for orthostatic hypotension are well-defined, various definitions for orthostatic hypertension are present in the medical literature. Regardless, the definitions of orthostatic hypotension or orthostatic hypertension use criteria based on changes in either systolic or diastolic blood pressure and combine the contributions of PP and MAP into a single phenotype. In addition, most definitions of orthostatic hypotension and orthostatic hypertension are based on relatively dramatic change, which would occur in a very small proportion of a community-based sample such as ours. Thus, by investigating more subtle variation in postural change of individual blood pressure measures, we were able to evaluate the relative contributions and varying relations of postural change in blood pressure measures with specific HMOD measures.

Upon standing, the initial fall in blood pressure initiates a compensatory autonomic response that seeks to restore blood pressure quickly in response to a change in baroreceptors activity, which is associated with an increase in sympathetic and decrease in parasympathetic activity. At the brachial artery (where blood pressure was measured), PP normally falls with a concurrent increase in MAP and DBP while SBP remains relatively stable with only a slight increase after standing from a supine position (4). Although MAP increases at the heart level, MAP at the brain level falls because the distance from heart level to brain creates a hydrostatic drop in brain perfusion pressure that exceeds the increase in MAP. Since myogenic tone is sensitive to both MAP and PP (35, 36) the combination of lower MAP and PP at the brain level promotes resistance vessel dilation that normally maintains cerebral perfusion. Previous clinical studies confirm that both exaggerated peripheral vasoconstriction and elevated heart rate contribute to excessive orthostatic blood pressure elevation (37, 38). We observed that an exaggerated postural increase in SBP and a paradoxical increase in PP were marginally to strongly associated with higher UACR—a marker of kidney damage. These data are consistent with findings from the Japan Morning Surge (JMS)-1 Study, where participants in the highest decile group of orthostatic SBP change (PP was not examined) also exhibited higher levels of B-type natriuretic peptide, which is a marker of renal and cardiac dysfunction (39). Recently, Sprick et al. observed that vascular α_1_-adrenergic sensitivity was enhanced in patients with chronic kidney disease (40). Taken together, these data support the hypothesis that α_1_-adrenergic response to orthostasis upon standing may have contributed to the observed relations of higher postural increase in SBP and PP with higher UACR in the current study. Similarly, several studies have shown that stimulation of α_1_-adrenergic receptors plays an important role in the development of cardiac hypertrophy (41). However, we did not observe significant relations of postural change in blood pressure measures with prevalent albuminuria examined as a dichotomous variable. We posit that among younger individuals with altered postural blood pressure responses, higher UACR may represent nascent kidney damage that may later progress to kidney disease. Alternatively, these data may indicate that only a moderate postural increase in PP and SBP contributes to higher afferent arteriolar resistance and minor endothelial damage of the renal glomeruli and tubules but does not lead to proteinuria or kidney disease. Similarly, while associations of higher postural change in SBP, MAP, and PP with higher LV mass index were marginally significant (*P* = 0.008–0.03), we did not observe significant relations of postural change in blood pressure measures with prevalent LV hypertrophy. Thus, baroreflex dysfunction may be insufficient to indicate early cardiac hypertrophy in younger individuals. Consistent with a previous FHS study (11), we did not observe significant relations of postural change in blood pressure measures with other subclinical brain MRI structural measures. Yet, interpretation of the current study should take into consideration not only the foregoing study but also the marginally statistically significant results in the context of multiple statistical testing. Additionally, longitudinal relations of altered baroreflex responses with subclinical kidney, heart, and brain damage warrant further study.

### Aortic stiffness, baroreflex, and hypertension-mediated organ damage

Aortic stiffening reduces impedance mismatch and wave reflection at the aorta-carotid interface. Age-related increase in aortic stiffness is accompanied by only minor changes in the stiffness of muscular arteries (9, 42). Stiffening of the proximal aorta, relative to the muscular and resistance arteries, increases the transmission of pressure pulsatility into the microcirculation, contributing to HMOD (9, 10). As high-flow, low-impedance organs, the brain and kidneys are particularly susceptible to damage (10). Contrary to Maillard et al. (43), we did not observe significant relations of aortic stiffness with brain fractional anisotropy. Unlike the aforementioned study, we did not assess brain HMOD relations stratified by white and gray matter and by specific brain regions, which may have contributed to differences between the current and foregoing studies for relations of fractional anisotropy outcomes. Additionally, the foregoing study included a subset of FHS Offspring participants, who were substantially older than the current sample. We observed a significant relation of higher aortic stiffness with higher brain free water content; this observation is consistent with prior work in a slightly older FHS sample (44). Free water reflects the water content that is not restricted by the surroundings (i.e., more free water indicates worse structural integrity) and is a robust indicator of subclinical cerebral injury (45, 46).

Higher aortic stiffness was associated with more subclinical kidney damage in men but not in women. Previous studies showed that progression of kidney damage and severity of kidney disease are greater in men (47, 48). These data suggest that sex-specific structural differences in the kidney and/or aorta and sex hormones may contribute to differences in the mechanisms by which abnormal aortic stiffness are associated with microvascular kidney damage and subsequent kidney disease. As mechanoreceptors within the aortic arch and carotid sinus, baroreceptors are sensitive to the elastic properties of the aorta. For example, aortic stiffening can limit the stretch activation of baroreceptors and attenuate the baroreflex in response to changes in systemic pressures (49). Furthermore, population-based studies have shown lower cardiovagal baroreflex sensitivity among individuals with elevated aortic stiffness (6, 50–52). Collectively, the foregoing investigations suggest a biological interaction of effects of aortic stiffness and baroreceptor activity on downstream organ structure and function. Consistent with this hypothesis, we observed that the extent of baroreflex dysfunction (assessed by postural change in blood pressure above vs. at/below the median value) modified the relation of aortic stiffness with kidney damage. Among participants with higher (above the median value) postural change in MAP and DBP, the effect of aortic stiffening on kidney damage was about 80% lower than participants with lower MAP and DBP orthostatic responses. Furthermore, among those with higher PP response (above the median value), the effect of aortic stiffening on kidney damage was about five times more severe compared with participants with lower postural change in PP. These data suggest that among individuals with the expected modest increase in MAP on standing, kidney damage associated with higher aortic stiffness is less severe, and conversely, that kidney damage is amplified in individuals with a blunted increase in MAP on standing. Normal orthostatic responses may contribute to higher resistance in the afferent arterioles in the kidneys, which may act as a mechanism to limit the pulsatile energy associated with higher aortic stiffness from penetrating the glomeruli (35). Conversely, abnormal orthostatic responses (i.e., higher PP but lower MAP upon standing) may exacerbate penetration of pulsatile energy into the kidneys and contribute to kidney damage (as evidenced by higher UACR).

### Clinical implications

Postural change in blood pressure is easily assessed in the clinic and may be used to refine CVD risk assessment. Elucidation of processes that are associated with variability in postural change in blood pressure may inform the development of novel prevention and therapeutic approaches. For example, the identification of early-onset subclinical indicators and key pathways associated with blunted or exaggerated orthostatic blood pressure response may identify individuals who are at risk for the development of HMOD or CVD. In the present study, we observed significant relations among middle-aged individuals; therefore, assessment of postural change in blood pressure measures may be relevant prior to older age before the clinical manifestation of high aortic stiffness, CVD, and HMOD. Additional studies are necessary to elucidate whether treating individuals with impairment of postural change in blood pressure could mitigate subclinical HMOD and prevent clinical disease.

### Limitations

The limitations of our study should be considered. We used a cross-sectional observational study design; thus, we cannot establish causation or temporal relations among postural changes in blood pressure and cfPWV with dichotomous and continuous indicators of HMOD. Longitudinal studies are needed to support the putative mechanisms due to differences in postural change in blood pressure and cfPWV. Presence of atrial fibrillation, overt heart failure, and pacemaker, while rare in our sample, may add to variability in aortic stiffness and blood pressure variables, which may lead to an underestimation of the strength of relations. Although we observed sex differences for the relation of cfPWV with UACR, we did not have the statistical power to resolve putative sex-specific associations for biological interaction of effects of aortic stiffness and baroreceptor activity on kidney damage (Supplementary Table 5). We did not assess reproducibility of postural changes in blood pressure measures. The variability would bias associations toward the null rather than contribute to false associations and may have contributed to some of the observed null relations given the use of relatively conservative Bonferroni-corrected α levels. cfPWV rises steeply after 50 years of age (42); thus, additional studies with an older sample are needed to establish whether these associations apply to older individuals. Echocardiographic parameters were assessed several years before hemodynamics assessment, which may bias those associations toward the null. In addition, our findings may not be generalizable to other ethnic groups since our cohort is mostly composed of participants of European descent. Consideration of these limitations should be balanced with acknowledgment of the strengths of our study. We have performed this analysis using a large, community-based sample with routine and standardized ascertainment of potential confounders and a comprehensive assessment of relations of postural blood pressure change with a battery of HMOD outcomes.

## Conclusion

In a sample of FHS participants, we investigated the inter-relations of postural change in blood pressure, aortic stiffness, and dichotomous and continuous indicators of HMOD. Our findings suggest that differences in baroreflex function are associated with subclinical kidney damage. Additionally, relations of higher aortic stiffness with greater kidney damage may be modified by associated differences in baroreflex function. Additional studies that further examine the inter-relations of aortic stiffness, baroreflex function, peripheral vascular function, and HMOD are warranted.

## Data availability statement

The datasets presented in this study can be found in online repositories. The procedure for requesting data from the FHS can be found at https://www.framinghamheartstudy.org/.

## Ethics statement

The studies involving human participants were reviewed and approved by the Institutional Review Board at Boston University School of Medicine. The patients/participants provided their written informed consent to participate in this study.

## Author contributions

LC, RV, and GM were responsible for the study’s concept and design. LC, JR, PM, AB, ML, CD, RV, SS, and GM were responsible for acquisition, analysis, or interpretation of data. LC drafted the manuscript. LC, ML, NH, RV, and GM critically revised the manuscript for important intellectual content. ML, JR, RV, SS, and GM provided administrative, technical, or material support. ML, RV, and GM supervised the study. All authors have read and approved the final manuscript.
