# Supplementary material for: Relations of postural change in blood pressure with hypertension-mediated organ damage in middle-aged adults of the Framingham heart study: A cross-sectional study
Source: Front Cardiovasc Med. 2022 Nov 1;9:1013876. doi: 10.3389/fcvm.2022.1013876 (PMC9663798; doi:10.3389/fcvm.2022.1013876)
Supplement: Supplementary file 1 [file Data_Sheet_1.docx]

Supplementary Material

# This supplementary material has been provided by the authors to give the readers additional information about their work.

**Supplementary Content:**

**Supplementary Figure.** Distributions for postural change in blood pressures.

**Supplementary Table 1**. Multivariable cross‐sectional relations between various cardiovascular disease risk factors and postural change in blood pressure measures: results from stepwise variable selection.

**Supplementary Table 2**. Multivariable-adjusted relations of postural change in blood pressure and presence of hypertension-mediated organ damage with additional adjustment for carotid-femoral pulse wave velocity.

**Supplementary Table 3.** Sex interactions for the relations of postural change in blood pressure and carotid-femoral pulse wave velocity with presence of hypertension-mediated organ damage.

**Supplementary Table 4.** Sex interactions for the relations of postural change in blood pressure and carotid-femoral pulse wave velocity with continuous measures of hypertension-mediated organ damage.

**Supplementary Table 5.** Effect modification by postural change in blood pressure for the relation of aortic stiffness with urinary albumin-creatinine ratio stratified by sex.

**Supplementary Table 6.** Matrix of Pearson correlation coefficients for postural change in heart rate and postural change in blood pressure measures.

**Supplementary Figure.** Distributions for postural change in blood pressures: (A) systolic blood pressure; (B) diastolic blood pressure; (C) mean arterial pressure; and (D) pulse pressure.
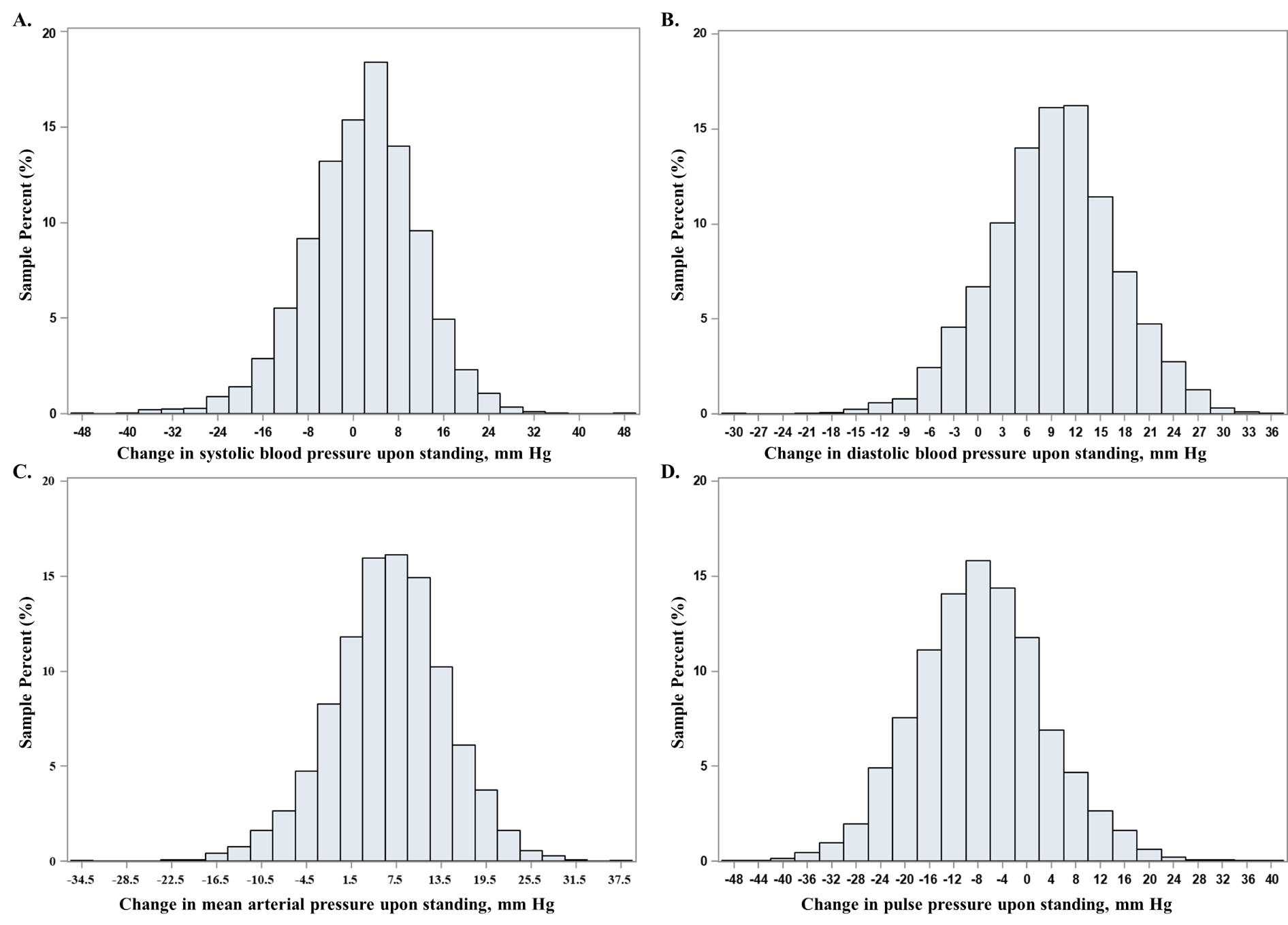


# Supplementary Tables

| **Supplementary Table 1.** Multivariable cross‐sectional relations between various cardiovascular disease risk factors and postural change in blood pressure measures: results from stepwise variable selection. | | | | | | | | |
| --- | --- | --- | --- | --- | --- | --- | --- | --- |
|  | **∆SBP** |  | **∆DBP** |  | **∆MAP** |  | **∆PP** |  |
| **CVD risk factor (N=3495)** | **Est. *β*±SE (*P*)** |  | **Est. *β*±SE (*P*)** |  | **Est. *β*±SE (*P*)** |  | **Est. *β*±SE (*P*)** |  |
| *Age | -0.03±0.02 (0.07) |  | -0.06±0.01 (<0.001) |  | -0.06±0.01 (<0.001) |  | 0.06±0.02 (<0.001) |  |
| *Sex | -1.67±0.33 (<0.001) |  | -0.31±0.27 (0.25) |  | 1.02±0.26 (<0.001) |  | -1.63±0.35 (<0.001) |  |
| *Corresponding supine BP | -0.24±0.01 (<0.001) |  | -0.31±0.01 (<0.001) |  | -0.31±0.01 (<0.001) |  | -0.31±0.01 (<0.001) |  |
| Body mass index | 0.45±0.03 (<0.001) |  | 0.12±0.03 (<0.001) |  | 0.32±0.02 (<0.001) |  | 0.35±0.03 (<0.001) |  |
| Heart rate | 0.08±0.02 (<0.001) |  | 0.03±0.01 (0.03) |  | 0.09±0.01 (<0.001) |  | 0.05±0.02 (0.004) |  |
| Total/HDL cholesterol | --- |  | --- |  | 0.30±0.12 (0.01) |  | --- |  |
| Alcohol consumption | 1.05±0.38 (0.006) |  | 1.22±0.31 (<0.001) |  | 1.10±0.29 (<0.001) |  | --- |  |
| Prevalent CVD | --- |  | --- |  | -2.33±1.04 (0.02) |  | --- |  |
| Current smoker | 1.29±0.53 (0.02) |  | --- |  | 0.90±0.40 (0.03) |  | 1.98±0.57 (<0.001) |  |
| Triglycerides | --- |  | 0.82±0.27 (0.003) |  | --- |  | --- |  |
| Prevalent diabetes | --- |  | -1.42±0.61 (0.02) |  | --- |  | --- |  |
| **Model R^2^** | **0.155** |  | **0.134** |  | **0.215** |  | **0.154** |  |
| ∆SBP, postural change in systolic blood pressure. ∆DBP, postural change in diastolic blood pressure. ΔMAP, postural change in mean arterial pressure. ∆PP, postural change in pulse pressure. HDL, high-density lipoprotein. CVD, cardiovascular disease. The regression coefficients (*β*) are per 1 standard deviation unit increase in continuous variables or presence of categorical variables. *Forced into the model. All coefficients are represented as standard deviation difference in blood pressure per standard deviation difference in continuous variables or presence of categorical variables. Candidate variables that did not enter or were not retained in the models included fasting glucose, HbA1c, hypertension treatment, hyperlipidemia treatment, and prevalent atrial fibrillation. | | | | | | | | |

| Supplementary Table 2. Multivariable-adjusted relations of postural change in blood pressure and presence of hypertension-mediated organ damage with additional adjustment for carotid-femoral pulse wave velocity. | | | | | | | | | | | | | |
| --- | --- | --- | --- | --- | --- | --- | --- | --- | --- | --- | --- | --- | --- |
|  | | **Left ventricle hypertrophy**  **N=3354** | |  | **Albuminuria**  **N=3488** | |  | **Covert brain infarcts**  **N=2096** | |  | **White matter hyperintensities**  **N=2095** | |  |
| **Vascular measures** | **OR (95% CI)** | | ***P*** |  | **OR (95% CI)** | ***P*** |  | **OR (95% CI)** | ***P*** |  | **OR (95% CI)** | ***P*** |  |
| **∆SBP** | 1.19 (1.02, 1.38) | | 0.02 |  | 1.12 (0.96, 1.31) | 0.15 |  | 1.21 (0.97, 1.52) | 0.09 |  | 1.03 (0.87, 1.23) | 0.7 |  |
| **∆DBP** | 1.04 (0.89, 1.22) | | 0.6 |  | 1.03 (0.88, 1.21) | 0.7 |  | 1.22 (0.97, 1.54) | 0.1 |  | 0.99 (0.83, 1.18) | 0.9 |  |
| **∆MAP** | 1.16 (0.98, 1.36) | | 0.08 |  | 1.11 (0.94, 1.31) | 0.2 |  | 1.27 (1.00, 1.62) | 0.047 |  | 1.05 (0.88, 1.26) | 0.6 |  |
| **∆PP** | 1.17 (1.01, 1.36) | | 0.04 |  | 1.10 (0.94, 1.29) | 0.2 |  | 1.05 (0.84, 1.32) | 0.7 |  | 1.05 (0.87, 1.25) | 0.6 |  |
| Odds ratios (OR) for each outcome are expressed per 1 standard deviation higher value of postural change in blood pressure. Bonferroni-adjusted *P* values (*P*=0.05/16=0.0031) were used to assess significance of associations. CI, confidence interval. **∆**SBP, postural change in systolic blood pressure. **∆**DBP, postural change in diastolic blood pressure. **∆**MAP, postural change in mean arterial pressure. **∆**PP, postural change in pulse pressure. All models adjusted for age, sex, corresponding supine blood pressure, body mass index, heart rate, total/high-density lipoprotein cholesterol ratio, alcohol consumption (past year), prevalent cardiovascular disease, current smoker, triglycerides, prevalent diabetes, and carotid-femoral pulse wave velocity. Covert brain infarcts and white matter hyperintensities models were further adjusted for total cranial volume and time between tonometry and brain magnetic resonance imaging. | | | | | | | | | | | | | |

| **Supplementary Table 3.** Sex interactions for the relations of postural change in blood pressure and carotid-femoral pulse wave velocity with presence of hypertension-mediated organ damage. | | | | |
| --- | --- | --- | --- | --- |
| **Sex interaction terms** | **Left ventricle hypertrophy**  **(N=3354)** | **Albuminuria**  **(N=3488)** | **Covert brain infarcts**  **(N=2096)** | **White matter hyperintensities**  **(N=2095)** |
|  | *P* Value | *P* Value | *P* Value | *P* Value |
| **∆SBP x female** | 0.60 | 0.54 | 0.91 | 0.17 |
| **∆DBP x female** | 0.07 | 0.07 | 0.88 | 0.12 |
| **∆MAP x female** | 0.25 | 0.10 | 0.90 | 0.17 |
| **∆PP x female** | 0.61 | 0.43 | 0.98 | 0.97 |
| **cfPWV x female** | 0.29 | **<0.001** | 0.83 | 0.68 |
| **∆**SBP, postural change in systolic blood pressure. **∆**DBP, postural change in diastolic blood pressure. **∆**MAP, postural change in mean arterial pressure. **∆**PP, postural change in pulse pressure. cfPWV, carotid-femoral pulse wave velocity. Bonferroni-adjusted *P* values (*P*=0.1/20=0.005) were used to assess significance of sex interaction terms. All models adjusted for age, sex, corresponding supine blood pressure, body mass index, heart rate, total/high-density lipoprotein cholesterol ratio, alcohol consumption (past year), prevalent cardiovascular disease, current smoker, triglycerides, and prevalent diabetes. Covert brain infarcts and white matter hyperintensities models were further adjusted for total cranial volume and time between tonometry and brain magnetic resonance imaging. | | | | |

| **Supplementary Table 4.** Sex interactions for the relations of postural change in blood pressure and carotid-femoral pulse wave velocity with continuous measures of hypertension-mediated organ damage. | | | | |
| --- | --- | --- | --- | --- |
| **Sex interaction terms** | **Left ventricular mass index**  **N=3354** | **UACR**  **N=3488** | **Free water**  **N=1994** | **Fractional anisotropy**  **N=1994** |
|  | *P* Value | *P* Value | *P* Value | *P* Value |
| **∆SBP x female** | 0.77 | 0.98 | 0.15 | 0.15 |
| **∆DBP x female** | 0.07 | 0.03 | 0.39 | 0.39 |
| **∆MAP x female** | 0.20 | 0.18 | 0.11 | 0.11 |
| **∆PP x female** | 0.50 | 0.10 | 0.43 | 0.43 |
| **cfPWV x female** | 0.56 | **<0.001** | 0.29 | 0.29 |
| **∆**SBP, postural change in systolic blood pressure. **∆**DBP, postural change in diastolic blood pressure. **∆**MAP, postural change in mean arterial pressure. **∆**PP, postural change in pulse pressure. cfPWV, carotid-femoral pulse wave velocity. Bonferroni-adjusted *P* values (*P*=0.1/20=0.005) were used to assess significance of sex interaction terms. All models adjusted for age, sex, corresponding supine blood pressure, body mass index, heart rate, total/high-density lipoprotein cholesterol ratio, alcohol consumption (past year), prevalent cardiovascular disease, current smoker, triglycerides, and prevalent diabetes. Covert brain infarcts and white matter hyperintensities models were further adjusted for total cranial volume and time between tonometry and brain magnetic resonance imaging. | | | | |

| Supplementary Table 5. Effect modification by postural change in blood pressure for the relation of aortic stiffness with urinary albumin-creatinine ratio stratified by sex. | | | | | |
| --- | --- | --- | --- | --- | --- |
| Vascular measures | **Women**  **N=1838** | |  | **Men**  **N=1650** | |
|  | **Est. *β*±SE** | ***P*** |  | **Est. *β*±SE** | ***P*** |
| cfPWV | -0.004±0.04 | 0.9 |  | 0.10±0.04 | 0.02 |
| Above median ΔSBP | 0.08±0.05 | 0.09 |  | 0.11±0.05 | 0.04 |
| *cfPWV x above median ∆SBP | **0.02±0.04** | **0.7** |  | **-0.04±0.05** | **0.4** |
|  |  |  |  |  |  |
| cfPWV | 0.09±0.04 | 0.2 |  | 0.19±0.04 | <0.001 |
| Above median ΔDBP | -0.05±0.05 | 0.3 |  | -0.01±0.05 | 0.8 |
| *cfPWV x above median ∆DBP | **-0.09±0.04** | **0.04** |  | **-0.12±0.05** | **0.02** |
|  |  |  |  |  |  |
| cfPWV | 0.05±0.04 | 0.2 |  | 0.15±0.04 | <0.001 |
| Above median ΔMAP | 0.07±0.05 | 0.2 |  | 0.11±0.05 | 0.04 |
| *cfPWV x above median ΔMAP | **-0.07±0.04** | **0.1** |  | **-0.07±0.05** | **0.2** |
|  |  |  |  |  |  |
| cfPWV | 0.003±0.04 | 0.9 |  | 0.05±0.04 | 0.25 |
| Above median ΔPP | 0.09±0.05 | 0.04 |  | 0.16±0.05 | 0.002 |
| *cfPWV x above median ∆PP | **0.05±0.04** | **0.3** |  | **0.12±0.05** | **0.02** |
| cfPWV, carotid-femoral pulse wave velocity. ∆SBP, postural change in systolic blood pressure. ∆DBP, postural change in diastolic blood pressure. ΔMAP, postural change in mean arterial pressure. ∆PP, postural change in pulse pressure. cfPWV, above median postural change in blood pressure measure (0 if ≤ median, 1 if > median), and the interaction term (cfPWV x above median change in blood pressure) were entered simultaneously as predictors in the models. The regression coefficients (*β*) are per 1 standard deviation unit increase in predictor value. *Bonferroni-adjusted *P* values (*P*=0.1/8=0.0125) were used to assess significance of interaction terms. All models adjusted for age, corresponding supine blood pressure, body mass index, heart rate, total/high-density lipoprotein cholesterol ratio, alcohol consumption (past year), prevalent cardiovascular disease, current smoker, triglycerides, and prevalent diabetes. | | | | | |

| **Supplementary Table 6.** Matrix of partial and Pearson correlation coefficients for postural change in heart rate and postural change in blood pressure measures (N=3495). | | | | | |
| --- | --- | --- | --- | --- | --- |
|  | **∆SBP** | **∆DBP** | **∆MAP** | **∆PP** |  |
| **Partial correlations*** | |  |  |  |  |
| **∆HR** | -0.19  <0.0001 | 0.14  <0.0001 | 0.004  0.81 | -0.29  <0.0001 |  |
| **Pearson correlations** | |  |  |  |  |
| **∆HR** | -0.12  <0.0001 | 0.17  <0.0001 | 0.08  <0.0001 | -0.25  <0.0001 |  |
| **∆SBP** |  | 0.30  <0.0001 | 0.72  <0.0001 | 0.71  <0.0001 |  |
| **∆DBP** |  |  | 0.76  <0.0001 | -0.46  <0.0001 |  |
| **∆MAP** |  |  |  | 0.11  <0.0001 |  |
| ∆HR, postural change in heart rate. ∆SBP, postural change in systolic blood pressure. ∆DBP, postural change in diastolic blood pressure. ∆MAP, postural change in mean arterial pressure. ∆PP, postural change in pulse pressure. Top value is *r* (unadjusted) or partial *r* (adjusted); bottom value is *P* in each cell. Bonferroni-adjusted *P* values (*P*=0.05/14=0.0036) were used to assess significance of correlations. *Partial correlations adjusted for age, sex, corresponding supine blood pressure, body mass index, supine heart rate, total/high-density lipoprotein cholesterol ratio, alcohol consumption, prevalent cardiovascular disease, current smoker, triglycerides, and prevalent diabetes. | | | | | |
